# Pollinator Foraging Adaptation and Coexistence of Competing Plants

**DOI:** 10.1371/journal.pone.0160076

**Published:** 2016-08-09

**Authors:** Tomás A. Revilla, Vlastimil Křivan

**Affiliations:** 1 Institute of Entomology, Biology Center, Czech Academy of Sciences, České Budějovice, Czech Republic; 2 Department of Mathematics and Biomathematics, Faculty of Science, University of South Bohemia, České Budějovice, Czech Republic; National Taiwan University, TAIWAN

## Abstract

We use the optimal foraging theory to study coexistence between two plant species and a generalist pollinator. We compare conditions for plant coexistence for non-adaptive vs. adaptive pollinators that adjust their foraging strategy to maximize fitness. When pollinators have fixed preferences, we show that plant coexistence typically requires both weak competition between plants for resources (e.g., space or nutrients) and pollinator preferences that are not too biased in favour of either plant. We also show how plant coexistence is promoted by indirect facilitation via the pollinator. When pollinators are adaptive foragers, pollinator’s diet maximizes pollinator’s fitness measured as the per capita population growth rate. Simulations show that this has two conflicting consequences for plant coexistence. On the one hand, when competition between pollinators is weak, adaptation favours pollinator specialization on the more profitable plant which increases asymmetries in plant competition and makes their coexistence less likely. On the other hand, when competition between pollinators is strong, adaptation promotes generalism, which facilitates plant coexistence. In addition, adaptive foraging allows pollinators to survive sudden loss of the preferred plant host, thus preventing further collapse of the entire community.

## Introduction

Et il se sentit très malheureux. Sa fleur lui avait raconté qu’elle était seule de son espèce dans l’univers. Et voici qu’il en était cinq mille, toutes semblables, dans un seul jardin!

Le Petit Prince, Chapitre XX – Antoine de Saint-Exupéry

The diversity and complexity of mutualistic networks motivate ecologists to investigate how they can remain stable and persistent over time. Mathematical models and simulations show that some properties of mutualistic networks (e.g., low connectance and high nestedness) make them more resistant against cascading extinctions [1], more likely to sustain large numbers of species [2], and more stable demographically [3]. However, simulations [4, 5] also indicate that mutualism increases competitive asymmetries, causing complex communities to be less persistent. These studies consider large numbers of species, parameters and initial conditions, making it difficult to understand the interplay between mutualisms (e.g., between plant and animal guilds) and antagonisms (e.g., resource competition between plants). These questions are easier to study in the case of community modules consisting of a few species only [6].

**Fig 1:**
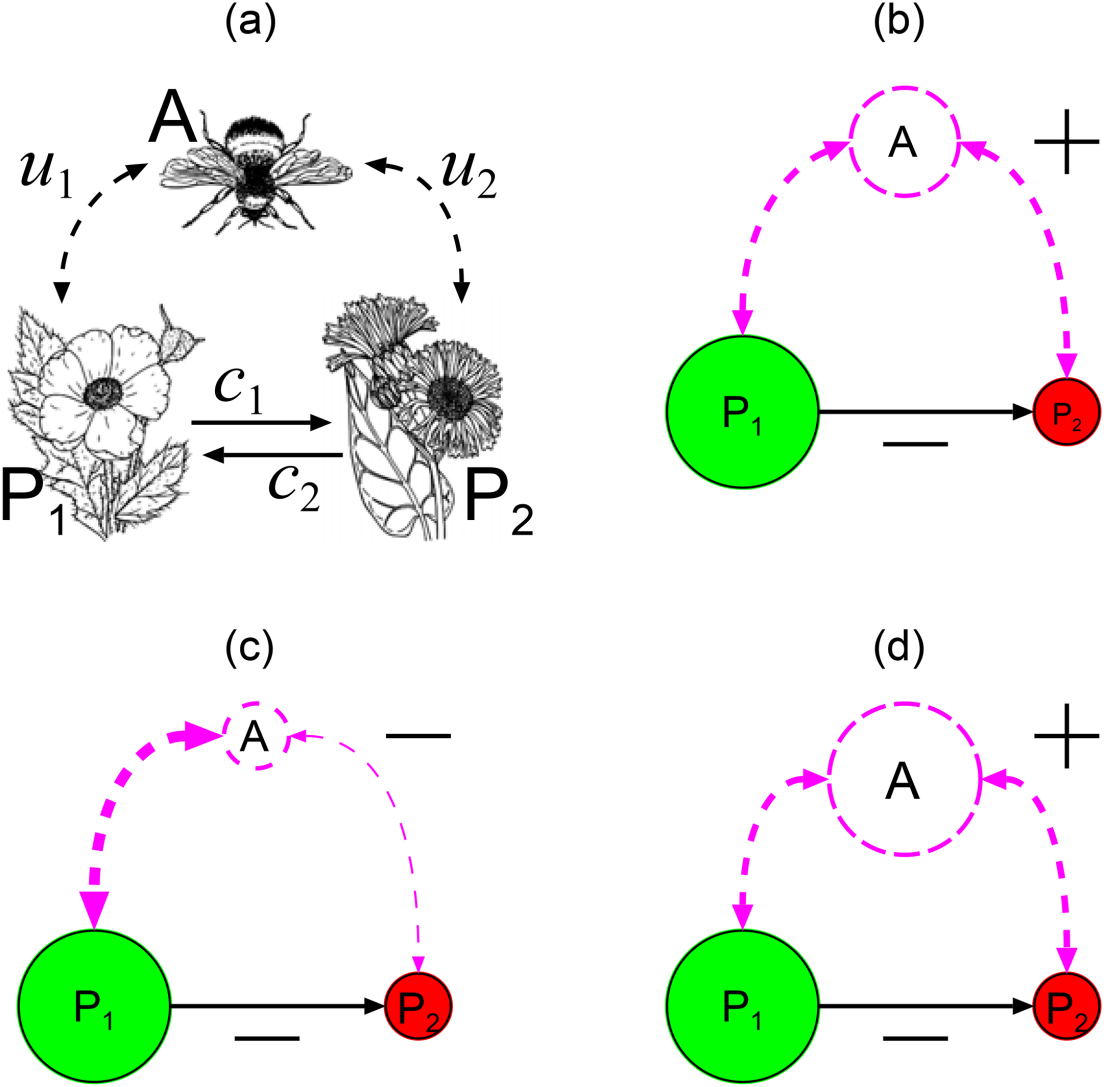
Community module consisting of plant 1 and 2, and pollinator A. (a) Plants affect each other directly (solid arrows) by competition for space or resources (*c*_1_, *c*_2_), and indirectly (dashed arrows) via shared pollinator with plant preferences *u*_1_ and *u*_2_. (b) When pollinator preferences are fixed and not too biased, a large density of plant 1 maintains a large pollinator density, which has an indirect positive effect on low density plant 2. In (c,d) pollinator preferences for plants are adaptive (dashed arrows change thickness). When pollinators are rare (c), preferences favour abundant plant 1, which results in a negative indirect effect on rare plant 2. When pollinators become abundant (d), competition between pollinators lead to balanced preferences, and the indirect effect on plant 2 becomes positive. The viability of plant 2 depends on the balance between indirect and direct effects. Image sources for panel (a) were taken from: https://openclipart.org.

In this article we consider a mutualistic module with two plant species and one pollinator species (Fig 1a). This module combines several direct and indirect interactions that are either density- or trait-mediated (sensu [7]). These include plant intra- and inter-specific competition (for e.g., space), plant competition for pollinator services, and pollinator intra-specific competition for plant resources (e.g., nectar). Some of these interactions depend on changes in population densities only (e.g., intra- and inter-specific plant competition), while the others depend also on individual morphological and behavioural traits. As some of them have positive and some of them negative effect on plant coexistence, it is difficult to predict their combined effects on species persistence and stability.

First, we will assume that pollinator preferences for plants are fixed. In this case, there is a negative effect of one plant on the other by direct competition and a positive indirect effect that is mediated by the shared pollinators, called facilitation [8–10]. As one plant population density increases, pollinator density increases too, which, in turn, increases pollination rate of the other plant (Fig 1b). This is an indirect interaction between plants that is mediated by changes in abundance of the pollinator (i.e., density mediated indirect interaction). Because facilitation has the opposite effect to direct plant competition (see Fig 1b) it is important to clarify under which situations the positive effect of facilitation prevails, and we study this question by using a mathematical model.

Second, we will assume that pollinator preferences are adaptive. We will assume that pollinator fitness is defined as the per capita pollinator population growth rate that depends on plant (that produce resources for pollinators) as well as on pollinator densities. First, pollinators benefit from nectar quality and nectar abundance (which correlates with plant population density). Second, pollinators compete for resources. This competition will play an important effect when pollinator population densities are high. A game theoretical approach to determine the optimal pollinator strategy is the Ideal Free Distribution (IFD) [11, 12]. This theory predicts that when pollinators are at low numbers, they will specialize on one plant only. As their population density will increase, they become generalists feeding on and pollinating both plants. This mechanism causes a negative effect of the preferred plant on the other plant, because when at low densities, pollinators will specialize on one plant only (Fig 1c). This is an example of a positive feedback where “the rich becomes richer and the poor get poorer”. Competition for pollinators is an example of a trait-mediated effect caused by pollinator behaviour. Pollinator specialization on one plant only is detrimental for the other plant. However, as pollinator population density will increase, competition for resources among pollinators will increase too [13], and the IFD predicts that they become generalists, which promotes plant coexistence (Fig 1d). Once again, combination of positive and negative effects between plants creates complicated feedbacks between population densities and pollinator behaviour that are impossible to disentangle without an appropriate mathematical model.

Our main goal is to study how pollinator preferences and plant competition affect plant coexistence. First, we study the dynamics of the plant–pollinator module when pollinator preferences are fixed. Second, we calculate the pollinator’s evolutionarily stable foraging strategy (ESS) at fixed plant and pollinator population densities, and we study plant coexistence assuming pollinators instantaneously track their ESS. This case corresponds to time scale separation where population dynamics operate on a slow time scale, while pollinator foraging preferences operate on a fast time scale. Finally, we consider the situation without time scale separation and we model preference dynamics with the replicator equation. Overall, we show that pollinator foraging adaptation has complex effects, sometimes equivocal, on plant coexistence. On the one hand pollinator adaptation increases competitive asymmetries among plants, promoting competitive exclusion. On the other hand competition for plant resources among pollinators promotes generalism over specialization, which can prevent the loss of pollination services for some plants and promote coexistence.

## Methods

### Mutualistic community model

Let us consider two plant species *P*_*i*_ (*i* = 1, 2) and one pollinator species *A* (Fig 1a). Plants produce resources *F*_*i*_ (*i* = 1, 2) such as nectar at a rate *a*_*i*_ per plant. Resources not consumed by the pollinator decrease with rate *w*_*i*_ (e.g., nectar can be re-absorbed, decay or evaporate). Resources are consumed by pollinators at rate *b*_*i*_ per resource per pollinator. Pollinator’s relative preferences for either plant are denoted by *u*_*i*_ with *u*_1_ + *u*_2_ = 1. Plant birth rates are proportional to the rate of pollen transfer that is concomitant with resource consumption. Thus, we assume that plant birth rates are proportional to pollinator resource consumption rates (*u*_*i*_ *b*_*i*_ *F*_*i*_ *A*) multiplied by conversion efficiency *r*_*i*_. Pollinator birth rates are proportional to resource consumption with corresponding conversion efficiency *e*_*i*_. Plants and pollinators die with the per capita mortality rate *m*_*i*_ (*i* = 1, 2) and *d*, respectively.

Assuming that plant resources equilibrate quickly with current plant and pollinator densities [14], i.e., *dF*_*i*_/*dt* = 0, plants and pollinator population dynamics are described by the following model (S1 Appendix)

$$\begin{array}{c} \frac{dP_{1}}{dt}=(\frac{r_{1}a_{1}u_{1}b_{1}A}{w_{1}+u_{1}b_{1}A}(1-\frac{P_{1}+c_{2}P_{2}}{K_{1}})-m_{1})P_{1} \end{array}$$ (1a)

$$\begin{array}{c} \frac{dP_{2}}{dt}=(\frac{r_{2}a_{2}u_{2}b_{2}A}{w_{2}+u_{2}b_{2}A}(1-\frac{P_{2}+c_{1}P_{1}}{K_{2}})-m_{2})P_{2} \end{array}$$ (1b)

$$\begin{array}{c} \frac{dA}{dt}=(\frac{e_{1}a_{1}u_{1}b_{1}P_{1}}{w_{1}+u_{1}b_{1}A}+\frac{e_{2}a_{2}u_{2}b_{2}P_{2}}{w_{2}+u_{2}b_{2}A}-d)A, \end{array}$$ (1c)

in which plant growth rates are regulated by competition for non-living resources (e.g., light, nutrients, space) according to the Lotka–Volterra competition model, where *c*_*j*_ is the negative effect of plant *j* on plant *i* relative to the effect of plant *i* on itself (i.e., competition coefficient), and *K*_*i*_ stands for the habitat carrying capacity [4]. Notice that plant growth rates saturate with pollinator density (e.g., $\frac{r_{1}a_{1}u_{1}b_{1}A}{w_{1}+u_{1}b_{1}A}$) and pollinator growth rates decrease due to intra-specific competition for plant resources (e.g., $\frac{e_{1}a_{1}u_{1}b_{1}P_{1}}{w_{1}+u_{1}b_{1}A}$) [15]. In this model plants and pollinators are obligate mutualists, i.e., without pollinators plants go extinct and without plants the pollinator goes extinct. We do not model facultative mutualism because this introduces additional factors (e.g. alternative pollinators, vegetative growth), which complicate the analysis of direct and indirect effects of the three species module.

### Fixed pollinator preferences

We start our analyses assuming that pollinator preferences for plants (*u*_1_ and *u*_2_ = 1 − *u*_2_) are fixed at particular values ranging from 0 to 1. This means that for *u*_1_ = 1 or 0 pollinators are plant 1 or plant 2 specialists, respectively, while for 0 < *u*_1_ < 1 they are generalists. Since model Eq (1a) is non-linear, analytical formulas for interior equilibria and corresponding stability conditions are out of reach. However, it is possible to obtain coexistence conditions by means of invasibility analysis. First, we obtain conditions for stable coexistence of a single plant-pollinator subsystem at an equilibrium. Second, we ask under what conditions the missing plant species can invade when the resident plant–pollinator subsystem is at the equilibrium. In particular, we are interested in the situation where each plant species can invade the other one, because this suggests coexistence of both plants and pollinators. Derivation of invasion conditions are provided in S1 Appendix.

In general, invasibility does not guarantee coexistence [16, 17]. Also, a failure to invade when rare does not rule out possibility of invasion success when the invading species is at large densities. For these reasons we complement our invasibility analysis by numerical bifurcation analysis using XPPAUT [18], and parameter values given in Table 1. While not empirical, the values fall within ranges typically employed by consumer–resource models (e.g., [19]). Plant-specific parameters are equal except for *e*_*i*_ and *u*_*i*_ (*i* = 1, 2). We assume that *e*_1_ > *e*_2_, i.e., plant 1 provides pollinators with higher energy when compared to plant 2.

**Table 1:** Parameters of Model (1a) and Eq (4).

Symbol	Description	Values/ranges
*r*_*i*_	conversion efficiency of pollination service to plant *i* seeds	0.1
*e*_*i*_	conversion efficiency of plant *i* resources to pollinator eggs	*e*_1_ = 0.2,*e*_2_ = 0.1
*m*_*i*_	plant *i* per capita mortality rate	0.01
*d*	pollinator per capita mortality rate	0.1
*c*_*i*_	competitive effect of plant *i* on plant *j*	*c*_*i*_ ≥ 0
*K*_*i*_	plant *i* habitat carrying capacity	*K*_*i*_ > 0
*a*_*i*_	plant *i* per capita resource production rate	0.4
*b*_*i*_	pollinator consumption rate of plant *i* resources	0.1
*w*_*i*_	plant *i* resource decay rate	0.25
*u*_*i*_	relative preference for plant *i*, where *u*_1_ + *u*_2_ = 1	0 to 1
*ν*	preference adaptation rate	*ν* ≥ 0

Plant 1 resources are more beneficial for the pollinator than those from plant 2 (*e*_1_ > *e*_2_), but all the other plant-specific parameters have the same values in order to facilitate comparisons.

### Adaptive pollinator preferences

When pollinators behave as adaptive foragers their plant preferences should maximize their fitness. The pay-off a pollinator gets when pollinating only plant *i* is defined as the per capita pollinator growth rate on that plant, i.e.,

$$\begin{array}{c} V_{1}\left(u_{1}\right)=\frac{e_{1}a_{1}b_{1}P_{1}}{w_{1}+u_{1}b_{1}A},\;\;V_{2}\left(u_{1}\right)=\frac{e_{2}a_{2}b_{2}P_{2}}{w_{2}+\left(1-u_{1}\right)b_{2}A}. \end{array}$$ (2)

We observe that these pay-offs depend both on plant and pollinator densities and on the pollinator distribution *u*_1_, i.e., they are both density and frequency dependent. Now let us consider fitness of a generalist mutant pollinator with strategy ${\tilde{u}}_{1}$. Its fitness is then defined as the average pay-off, i.e.,

$$\begin{array}{c} W\left({\tilde{u}}_{1},u_{1}\right)={\tilde{u}}_{1}V_{1}\left(u_{1}\right)+\left(1-{\tilde{u}}_{1}\right)V_{2}\left(u_{1}\right)=V_{2}\left(u_{1}\right)+(V_{1}\left(u_{1}\right)-V_{2}\left(u_{1}\right)){\tilde{u}}_{1}. \end{array}$$ (3)

Using this fitness function we will calculate the evolutionarily stable strategy [20, 21] of pollinator preferences at current plant and pollinator densities. When pollinators adjust their preferences very fast as compared to changes in population densities, we will use the ESS together with population dynamics Eq (1a) to model effects of pollinator plasticity on population dynamics. This approach corresponds to time scale separation where population densities (plants and animals) change very slowly compared to pollinator adaptation. We are also interested in the situation when the two time scales are not separated, but pollinators foraging preferences still tend to the ESS. In these cases we use the replicator equation [21] to model dynamics of pollinator preferences *u*_1_ for plant 1 (*u*_2_ = 1 − *u*_1_)

$$\begin{array}{c} \frac{du_{1}}{dt}=\nu u_{1}\left(1-u_{1}\right)(\frac{e_{1}a_{1}b_{1}P_{1}}{w_{1}+u_{1}b_{1}A}-\frac{e_{2}a_{2}b_{2}P_{2}}{w_{2}+\left(1-u_{1}\right)b_{2}A}), \end{array}$$ (4)

where *ν* ≥ 0 is the adaptation rate. Eq (4) assumes that pollinator’s preferences evolve towards a higher energy intake and its equilibrium coincides with the ESS. Thus, if pollinators obtain more energy when feeding on plant 1, preferences for plant 1 increases. When *ν* ≥ 1, adaptation is as fast as population dynamics or faster. This describes plastic pollinators that track changing flower densities very quickly (i.e., within an individual life-span). This is the case when adaptation is a behavioural trait. In fact, for *ν* tending to infinity pollinators adopt the ESS instantaneously. Adaptation can also involve morphological changes requiring several generations (i.e., evolution). In that case *ν* < 1, and adaptation lags behind population dynamics (i.e., changes in preferences require more generations). And the *ν* = 0 case applies to non-adaptive pollinators. We remark that perfect specialization on plant 1 or plant 2 correspond to the equilibrium *u*_1_ = 1 or *u*_1_ = 0, respectively.

Using Model (1a) and replicator Eq (4), we simulate the effects of pollinator adaptation and plant direct inter-specific competition on coexistence. We consider four common inter-specific competition coefficients: *c*_1_ = *c*_2_ = *c* = 0, 0.4, 0.8, and 1.2, and four adaptation rates: *ν* = 0, 0.1, 1 and *ν* = ∞. Level *ν* = 0 extends our analysis for non-adaptive pollinators (fixed preferences) beyond invasion conditions. Level *ν* = 0.1 implies slow evolutionary adaptation, like in adaptive dynamics [22]. At *ν* = 1 adaptation is as fast as demography, i.e., pollinators adapt during their lifetime. For *ν* = ∞ adaptation is infinitely fast when compared to population densities and preferences are given by the ESS.

Community dynamics and the dynamics of pollinator preferences can be sensitive to initial conditions. There are four degrees of freedom for the initial conditions (*P*_1_, *P*_2_, *A* and *u*_1_ at *t* = 0). We reduce this number to two degrees of freedom. First, we vary *P*_1_(0) from 0 to *K* in 100 steps while *P*_2_(0) = *K* − *P*_1_(0), where *K* = *K*_1_ = *K*_2_ = 50 is the common carrying capacity. The choice *K* = 50 is high enough to avoid pollinator extinction due to the Allee effect in the majority of the simulations. Second, we consider two scenarios:

**Scenario I**: Initial pollinator density *A*(0) varies from 0 to 50 in 100 steps and initial pollinator preference is equal to the ESS.
**Scenario II**: Initial pollinator preference *u*_1_(0) varies from 0.001 to 0.999 in 100 steps [0.001, 0.01, 0.02, …, 0.98, 0.99, 0.999] and initial pollinator density is kept at *A*(0) = 2.

Scenario **I** assumes that pollinators preferences are at the ESS for given initial plant and pollinator densities, with an exception when the ESS is 0 or 1 in which case we perturb it to *u*_1_ = 0.001 or *u*_1_ = 0.999. This is necessary because the replicator Eq (4) does not consider mutations that may allow specialists to evolve towards generalism.

Scenarios **I** and **II** complement each other. In both of them initial plant composition (*P*_1_: *P*_2_) influences the outcome. For scenario **II** we also used *A*(0) = 50, but we did not find important qualitative differences. Thus, for both scenarios we simulate Models (1a) and (4) with 100 × 100 = 10^4^ different initial conditions. This systematic approach allows us to delineate boundaries between plant coexistence and extinction regions. Models (1a) and (4) is integrated (Runge–Kutta 4th, with Matlab [23]) with the rest of the parameters taken from Table 1. A plant is considered extinct if it attains a density less than 10^−6^ after time *t* = 20000.

## Results

### Fixed preferences

System (1a) models obligatory mutualism between plants and pollinators. Plants cannot grow in absence of pollinators and pollinators cannot reproduce without plants. Thus, the trivial equilibrium at which all three species are absent (*P*_1_ = *P*_2_ = *A* = 0) is always locally asymptotically stable [24, 25], because when at low population densities, pollinators cannot provide enough pollination services to plants that will die and, similarly, when at low densities, plants do not provide enough nectar to support pollinators.

By setting *dP*_1_/*dt* = *dA*/*dt* = 0 with *P*_1_ > 0, *P*_2_ = 0, *A* > 0 in Eq (1a) and (1c), non-trivial plant 1–pollinator equilibria are

$$\begin{array}{ccc} P_{1\pm } & = & \frac{b_{1}e_{1}K_{1}\left(a_{1}r_{1}-m_{1}\right)u_{1}+dr_{1}w_{1}\pm \sqrt{D_{1}}}{2a_{1}b_{1}e_{1}r_{1}u_{1}}\\ A_{1\pm } & = & \frac{b_{1}e_{1}K_{1}\left(a_{1}r_{1}-m_{1}\right)u_{1}-dr_{1}w_{1}\pm \sqrt{D_{1}}}{2b_{1}dr_{1}u_{1}}, \end{array}$$ (5)

where *D*_1_ = −4*b*_1_ *de*_1_ *K*_1_ *m*_1_ *r*_1_ *u*_1_ *w*_1_ + (*b*_1_ *e*_1_ *K*_1_(*m*_1_ − *a*_1_ *r*_1_)*u*_1_ + *dr*_1_ *w*_1_)^2^. These two equilibria are feasible (positive) if *a*_1_ *r*_1_ > *m*_1_ and *D*_1_ > 0. The first is a growth requirement: if not met, even an infinite number of specialized pollinators (with *u*_1_ = 1) cannot prevent plant 1 extinction. The second condition is met when pollinator preference for plant 1 (*u*_1_) is above a critical value

$$\begin{array}{c} u_{1a}=\frac{dr_{1}w_{1}}{b_{1}e_{1}{\left(\sqrt{a_{1}r_{1}}-\sqrt{m_{1}}\right)}^{2}K_{1}}. \end{array}$$ (6)

By symmetry, there are two non-trivial plant 2–pollinator equilibria (*P*_2±_, *A*_2±_). They are feasible if *a*_2_ *r*_2_ > *m*_2_ and *D*_2_ > 0 (*D*_2_ is like *D*_1_ with interchanged sub-indices). The second condition is met when pollinator preferences for plant 2 are strong enough (i.e., preferences for plant 1 are weak enough) so that *u*_1_ is below a critical value *u*_1*b*_

$$\begin{array}{c} u_{1b}=1-\frac{dr_{2}w_{2}}{b_{2}e_{2}{\left(\sqrt{a_{2}r_{2}}-\sqrt{m_{2}}\right)}^{2}K_{2}}. \end{array}$$ (7)

In both cases the equilibrium that is closer to the origin ((*P*_1−_, *A*_1−_) when plant 2 is missing and (*P*_2−_, *A*_2−_) when plant 1 is missing) is unstable. This instability indicates critical threshold densities. When plant *i* and pollinator densities are above these thresholds, coexistence is possible. Otherwise, the system converges on the extinction equilibrium mentioned before. This is a mutualistic Allee effect [26, 27].

The equilibrium that is farther from the origin ((*P*_1+_, *A*_1+_) when plant 2 is missing and (*P*_2+_, *A*_2+_) when plant 1 is missing) will be called the resident equilibrium. Resident equilibria are stable with respect to small changes in resident plant and pollinator densities, but may be unstable against invasion of small densities of the missing plant species. In the case of the plant 1–pollinator equilibrium (*P*_1+_, *A*_1+_), plant 2 invades (i.e., achieves a positive growth rate when rare) if the competitive effect of plant 1 on plant 2 (*c*_1_), is smaller than

$$\begin{array}{c} \alpha \left(u_{1}\right)=\frac{a_{1}b_{1}r_{1}u_{1}K_{2}(2b_{2}u_{2}e_{1}K_{1}m_{1}w_{1}\left(a_{2}r_{2}-m_{2}\right)-m_{2}w_{2}(b_{1}e_{1}K_{1}u_{1}\left(a_{1}r_{1}-m_{1}\right)-dr_{1}w_{1}-\sqrt{D_{1}}))}{a_{2}b_{2}r_{2}u_{2}K_{1}m_{1}w_{1}(b_{1}e_{1}K_{1}u_{1}\left(a_{1}r_{1}-m_{1}\right)+dr_{1}w_{1}+\sqrt{D_{1}})}, \end{array}$$ (8)

whereas plant 1 invades the plant 2–pollinator equilibrium (*P*_2+_,*A*_2+_) if the competitive effect of plant 2 on plant 1 (*c*_2_), is smaller than

$$\begin{array}{c} \beta \left(u_{1}\right)=\frac{a_{2}b_{2}r_{2}u_{2}K_{1}(2b_{1}u_{1}e_{2}K_{2}m_{2}w_{2}\left(a_{1}r_{1}-m_{1}\right)-m_{1}w_{1}(b_{2}e_{2}K_{2}u_{2}\left(a_{2}r_{2}-m_{2}\right)-dr_{2}w_{2}-\sqrt{D_{2}}))}{a_{1}b_{1}r_{1}u_{1}K_{2}m_{2}w_{2}(b_{2}e_{2}K_{2}u_{2}\left(a_{2}r_{2}-m_{2}\right)+dr_{2}w_{2}+\sqrt{D_{2}})}. \end{array}$$ (9)

**Fig 2:**
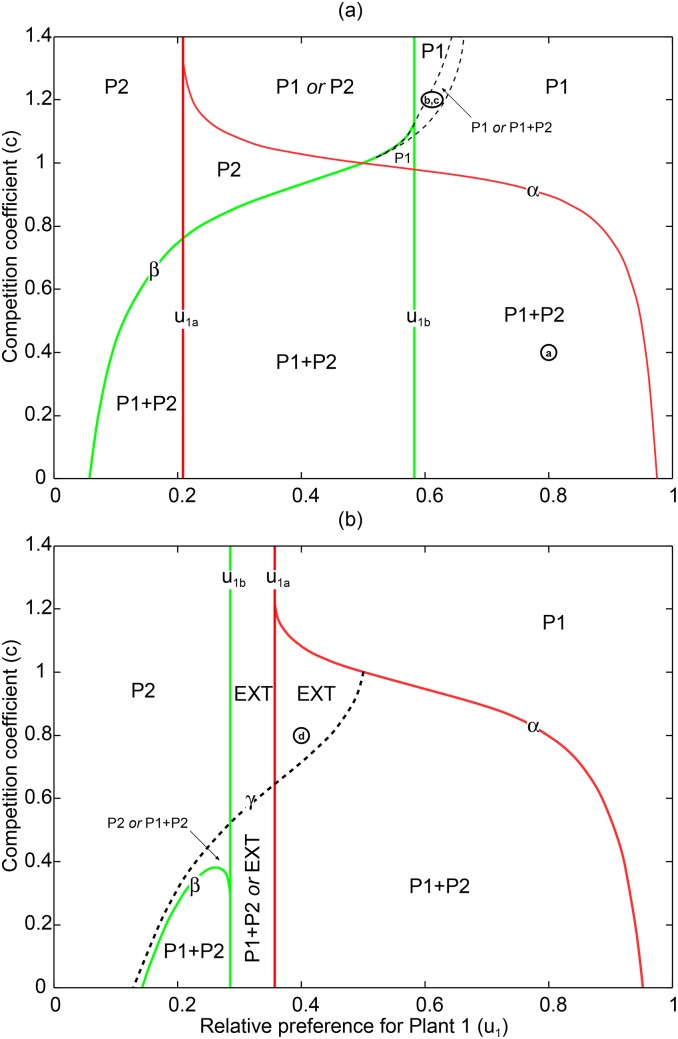
Interaction outcomes as a function of competition strength and fixed pollinator preferences. Solid lines (coloured, found analytically) determine regions where single plant equilibria exist and whether they can be invaded or not. Dashed lines (in black, found numerically, like *γ*) determine outcomes that cannot be predicted by invasibility analysis. Plant 2 can invade Plant 1 in the region between the red vertical line *u*_1*a*_ and the red curve *α*. Plant 1 can invade plant 2 in the region between the green curve *β* and the green vertical line *u*_1*b*_. The final composition of the community is indicated by P1 = plant 1 wins, P2 = plant 2 wins, P1 + P2 = coexistence, EXT = extinction of all species; the “*or*” separator indicates that the outcome depends on the initial conditions. Parameters from Table 1, with (a) *K*_*i*_ = 60 and (b) *K*_*i*_ = 35 (i.e., above and below critical K* = 37.5, see Eq (10)). Representative dynamics for parameter combinations at ⓐ, ⓑ, ⓒ and ⓓ are illustrated in corresponding panels of Fig 3.

Functions *α*(*u*_1_) and *β*(*u*_1_) are real when *D*_1_ > 0 and *D*_2_ > 0, respectively. In other words, invasibility only makes sense when the plant 1–pollinator resident equilibrium exists (*u*_1_ > *u*_1*a*_) or, when the plant 2–pollinator resident equilibrium exists (*u*_1_ < *u*_1*b*_), respectively. The graphs of Eqs (6), (7), (8) and (9) divide the pollinator preference–competition parameter space into several regions (Fig 2 where *c* = *c*_1_ = *c*_2_). Notice that because *α* and *β* are only feasible to the right of *u*_1*a*_ and to the left of *u*_1*b*_, respectively, their graphs may or may not intersect depending on the position of *u*_1*a*_ and *u*_1*b*_ (cf. panel a vs. b). We show this by setting a common plant carrying capacity *K* = *K*_1_ = *K*_2_ and making it larger or smaller than a critical value (S1 Appendix)

$$\begin{array}{c} K^{*}=\frac{(b_{1}e_{1}r_{2}w_{2}{\left(\sqrt{a_{1}r_{1}}-\sqrt{m_{1}}\right)}^{2}+b_{2}e_{2}r_{1}w_{1}{\left(\sqrt{a_{2}r_{2}}-\sqrt{m_{2}}\right)}^{2})d}{b_{1}b_{2}e_{1}e_{2}{\left(\sqrt{a_{1}r_{1}}-\sqrt{m_{1}}\right)}^{2}{\left(\sqrt{a_{2}r_{2}}-\sqrt{m_{2}}\right)}^{2}}. \end{array}$$ (10)

Productive environments (*K* > K*) support coexistence of both plant–pollinator resident equilibria for intermediate pollinator preferences. This is not so in unproductive environments (*K* < K*), where resident equilibria occur within separated ranges of pollinator preferences (see below).

**Fig 3:**
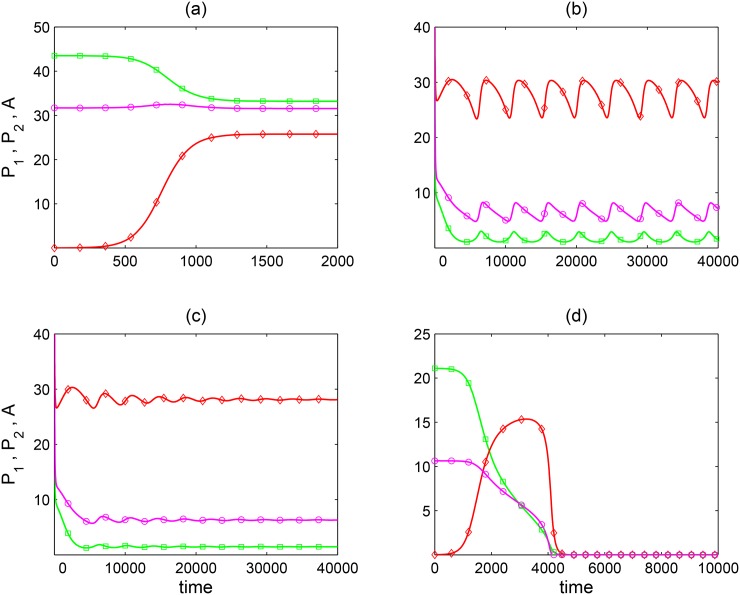
Model (1a) dynamics with fixed pollinator preferences. Population densities are represented by: green squares = plant 1, red diamonds = plant 2 and pink circles = pollinator. Panels (a) *K*_*i*_ = 60, *u*_1_ = 0.8, *c*_*i*_ = 0.4, (b) *K*_*i*_ = 60, *c*_*i*_ = 1.2, *u*_1_ = 0.605, (c) *K*_*i*_ = 60, *c*_*i*_ = 1.2, *u*_1_ = 0.607 and (d) *K*_*i*_ = 35, *u*_1_ = 0.4, *c*_*i*_ = 0.8 correspond to positions of points ⓐ, ⓑ, ⓒ and ⓓ in Fig 2. Other parameters as in Table 1.

First, we assume a high plant carrying capacity satisfying *K* > K*. Then *u*_1*a*_ < *u*_1*b*_, and *α*(*u*_1_) and *β*(*u*_1_) intersect like in Fig 2a. This leads to several plant invasion scenarios. We start with preferences satisfying *u*_1*a*_ < *u*_1_ < *u*_1*b*_. Such intermediate pollinator preferences allow each species to coexist with the pollinator at a stable equilibrium. If competition is weak enough (see the region denoted as “P1 + P2” in Fig 2a), the missing plant can invade the resident plant–pollinator equilibrium which leads to both plant coexistence. In contrast, if competition is strong enough (see the region denoted as “P1 *or* P2” in Fig 2a), the missing plant cannot invade. Thus, either plant 1 or plant 2 wins depending on the initial conditions (i.e., the resident plant that establishes first wins). In between these two outcomes of mutual invasion and mutual exclusion, there are two wedge-shaped regions (see regions denoted as “P1”, and “P2” in Fig 2a). In the right (left) region plant 1 (plant 2) invades and replaces plant 2 (plant 1) but not the other way around. The outcomes in the regions of Fig 2a that are either to the left of *u*_1*a*_, or to the right of *u*_1*b*_ are very different, because whether the missing plant can invade or not when rare depends entirely on facilitation by the resident plant. Indeed, let us consider the region of the parameter space in Fig 2a to the right of the vertical line *u*_1*b*_ and below the curve *α*. In this region (denoted by “P1 + P2”) pollinator preference for plant 2 is so low that plant 2 alone cannot support pollinators at a positive density. It is only due to presence of plant 1 that allows plant 2 survival through facilitation (Fig 3a). Indeed, when plant 1 is resident, it increases pollinator densities to such levels that allow plant 2 to invade. In other words, plant facilitation due to shared pollinators widens plant niche measured as the range of pollinator preferences at which the plant can survive at positive densities. When inter-specific plant competition is too high (see the region above the curve *α* and to the right of *u*_1*b*_) plant 2 cannot invade. Similarly, when pollinator preferences for plant 1 are too low (i.e., to the left of the line *u*_1*a*_), coexistence relies on facilitation provided by plant 2 (resident) to plant 1 (invader) and on plant competition being not too strong (below curve *β*); if competition is too strong (above *β*) plant 1 cannot invade.

Second, we assume low plant carrying capacity satisfying *K* < K*. Then *u*_1*a*_ > *u*_1*b*_, and *α*(*u*_1_) and *β*(*u*_1_) never intersect (Fig 2b) in the positive quadrant of the parameter space. For intermediate pollinator preferences satisfying *u*_1*b*_ < *u*_1_ < *u*_1*a*_ coexistence by invasion of the rare plant is not possible. The reason is that in this region neither plant 1, nor plant 2, can coexist with pollinators. So, there is no resident system consisting of one plant and pollinators that could be invaded by the missing rare plant. In regions to the left of *u*_1*b*_ plant 2 coexists with pollinators and to the right of *u*_1*a*_ plant 1 coexists with pollinators at a stable equilibrium and invasion conditions for the missing plant when rare are similar to the case where *K* > K*. Once again, in these regions coexistence of both plants can be achieved because of the resident plant facilitates the other plant invasion. The important prediction of this invasion analysis is that the density mediated indirect interaction between plants through the shared pollinator, i.e., plant facilitation, increases the set of parameter values for which coexistence of both plants is possible.

Although invasion analysis proves to be very useful when analysing Model (1a), it does not answer the question whether there are some other attractors that cannot be reached by invasion of the missing species when rare. Using numerical bifurcation software (XPPAUT [18]), we found additional outcomes not predicted by invasibility analysis. When *K* > K* (Fig 2a) invasibility analysis predicts that plant 2 cannot grow when rare for strong inter-specific plant competition when *c* > *α*. However, our numerical analysis shows that it is still possible for plant 2 to invade provided its initial population density is large enough. The community dynamics then either oscillate along a limit cycle (Fig 3b), or converge to a stable equilibrium (Fig 3c). Such behaviour was observed in the region denoted by “P1 *or* P1 + P2” of Fig 2a. This shows that Model (1a) has multiple attractors (including a limit cycle). The right dashed boundary of that region corresponds to a fold bifurcation where a locally stable interior equilibrium merges with an unstable equilibrium and disappears for higher values of *u*_1_. Between the two dashed curves there is another Hopf bifurcation curve (not shown in Fig 2a) where the interior equilibrium looses its stability and a limit cycle emerges. As preference for plant 1 decreases towards the left dashed boundary, the amplitude of the limit cycle tends to infinity.

When *K* < K* we found a curve *γ*(*u*_1_) that further divides the parameter space (Fig 2b). For the intermediate pollinator preferences (*u*_1*b*_ < *u*_1_ < *u*_1*a*_) where neither plant can be a resident, and below *γ* curve (“P1 + P2 or EXT”), coexistence is achievable if both plants and the pollinator are initially at high enough densities. This is an extreme example of plant facilitation. If combined plant abundances are not large enough, then both plants and the pollinator go extinct as already predicted by the invasion analysis. Also, if one plant species is suddenly removed, extinction of pollinator and the other plant follows. Above the *γ* curve, plant competition is too strong to allow any coexistence and the outcome is always global extinction (“EXT”). When preference for plant 1 is low (*u*_1_ < *u*_1*b*_), the *γ* curve is slightly above the *β* curve so that the possible coexistence region is slightly larger than the coexistence region obtained by invasion of the rare plant (“P2 *or* P1 + P2”). However, plant coexistence in the region between the two curves depends on the initial density of plant 1: if *P*_1_(0) is very low, plant 2 wins as predicted by the invasion analysis, but if *P*_1_(0) is large enough, plant 1 will invade and coexist at an interior equilibrium with plant 2. In the opposite situation, where preference for plant 1 is very high (*u*_1_ > *u*_1*a*_), *γ* divides the region where plant 2 can invade (assuming *c* < *α*) as follows. Below *γ*, competition is weak and plant 2 invasion is followed by stable coexistence thanks to resident facilitation. Above *γ*, competition is strong and plant 2 invasion causes plant 1 extinction followed by plant 2 extinction. This is because pollinator preference for plant 1 is too strong (*u*_1_ > *u*_1*b*_) which does not allow pollinators to survive on plant 2. Thus, invasion by plant 2 leads to global extinction (“EXT”). Fig 3d shows an example of such global extinction caused by invasion. Once again, invasion of plant 2 is possible due to facilitation by plant 1. As plant 2 invades, it has also an indirect positive effect on plant 1 through facilitation. But this positive effect does not outweigh the direct negative effect plant 2 has on plant 1 due to direct competition for resources. Apart from this case of global extinction caused by invasion, numerical analysis with parameters from Table 1 confirms predictions of our invasion analysis that in the case where one or both equilibria with one plant missing exist(s), the invasibility conditions *c* < *α* and *c* < *β* predict the existence of a locally stable interior equilibrium at which both plants coexist with the pollinator.

### Adaptive preferences

#### Evolutionarily stable strategy and time scale

We calculate the evolutionarily stable strategy for fitness defined by Eq (3). At the interior (i.e., generalist) behavioural equilibrium the two pay-offs Eq (2) must be the same, i.e., *V*_1_ = *V*_2_, which yields

$$\begin{array}{c} u_{1}^{*}\left(P_{1},P_{2},A\right)=\frac{e_{1}a_{1}P_{1}}{e_{1}a_{1}P_{1}+e_{2}a_{2}P_{2}}+\frac{w_{2}e_{1}a_{1}b_{1}P_{1}-w_{1}e_{2}a_{2}b_{2}P_{2}}{b_{1}b_{2}\left(e_{1}a_{1}P_{1}+e_{2}a_{2}P_{2}\right)A}, \end{array}$$ (11)

provided $u_{1}^{*}$ is between 0 and 1. If *V*_1_(*u*_1_)>*V*_2_(*u*_1_) for all *u*_1_, the ESS is $u_{1}^{*}=1$ and if *V*_1_(*u*_1_)<*V*_2_(*u*_1_) for all *u*_1_, the ESS is $u_{1}^{*}=0$. Because

$$\begin{array}{c} W\left(u_{1}^{*},u_{1}\right)-W\left(u_{1},u_{1}\right)=\frac{(Ab_{1}b_{2}{\left(a_{2}e_{2}P_{2}u_{1}-a_{1}e_{1}P_{1}\left(1-u_{1}\right)\right)+a_{2}b_{2}e_{2}P_{2}w_{1}-a_{1}b_{1}e_{1}P_{1}w_{2})}^{2}}{Ab_{1}b_{2}\left(a_{1}e_{1}P_{1}+a_{2}e_{2}P_{2}\right)\left(Ab_{1}u_{1}+w_{1}\right)\left(Ab_{2}\left(1-u_{1}\right)+w_{2}\right)}>0 \end{array}$$

the interior strategy $u_{1}^{*}$ is also resistant to mutant invasions [20], i.e., $W\left(u_{1}^{*},u_{1}\right)>W\left(u_{1},u_{1}\right)$ for all strategies $u_{1}\neq u_{1}^{*}$. Thus $u_{1}^{*}$ is the ESS [21]. We remark that in the ecological literature such an ESS strategy has also been called the Ideal Free Distribution [11, 12].

**Fig 4:**
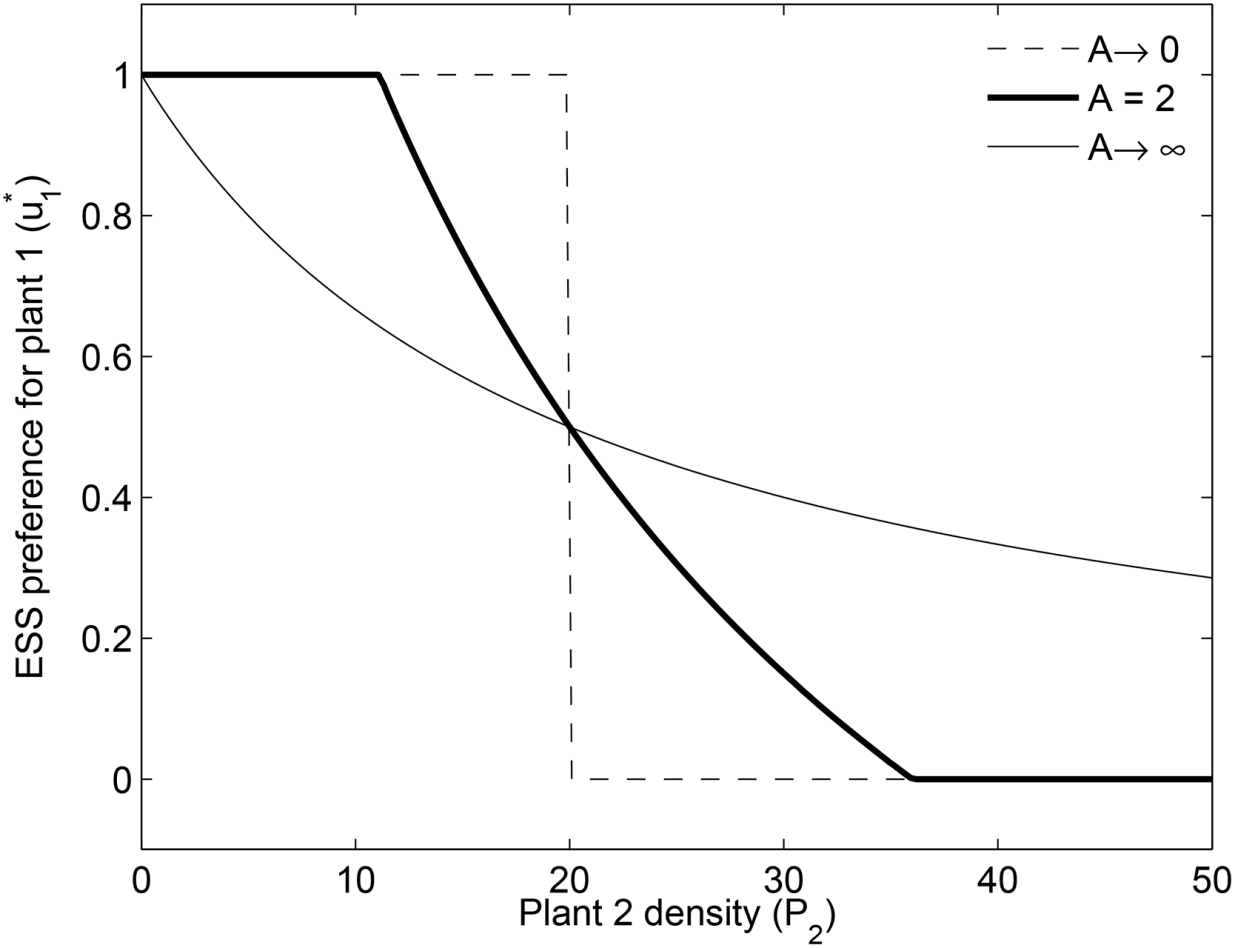
Evolutionarily stable preference for plant 1 as plant 2 density increases. Plant 1 density is fixed at *P*_1_ = 10 in Eq (11). At very low pollinator density preference switches abruptly (dashed line) from 1 to 0. At very high pollinator density the decline is continuous (thin line). Intermediate pollinator densities (thick line) cause a combined pattern with switching between specialisation (horizontal segments) and generalism (decreasing segment). Values of parameters are those given in Table 1.

It follows from Eq (11) that as pollinator densities increase, $u_{1}^{*}$ tends to *e*_1_ *a*_1_ *P*_1_/(*e*_1_ *a*_1_ *P*_1_ + *e*_2_ *a*_2_ *P*_2_), i.e., pollinators tend towards generalism, with relative preferences reflecting differences in resource supply rates and quality. This is because at higher pollinator densities fitness decreases due to intra-specific competition among pollinators for plant resources, which is compensated for by interacting with the less profitable plant. In contrast, when pollinator densities become very low, $u_{1}^{*}$ as a function of plant 2 density approximates a step function (Fig 4). In this latter case pollinators specialize either on plant 1 or on plant 2 and the switch between these two possibilities is very sharp. In this case competition between pollinators is so weak, that pollinators can afford to ignore the less profitable plant.

Eq (11) when combined with population dynamics Eq (1a) describes the situation where pollinator preferences instantaneously track population numbers. This situation corresponds to complete time scale separation between behavioural and population processes. When the assumption of time scale separation is relaxed, we show that the rate *ν* with which pollinator preferences change in Eq (4) has important effects on plant coexistence.

**Fig 5:**
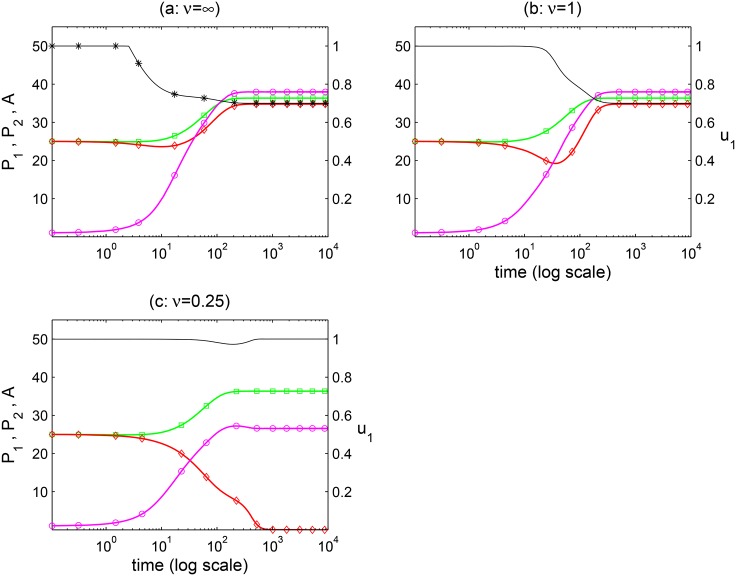
Model (1a) dynamics with pollinator adaptive preferences. Population densities (units in left axes) are represented by: green squares = plant 1, red diamonds = plant 2, pink circles = pollinator. Pollinator preference for plant 1 (*u*_1_, units in right axes) is represented by the black line. Initial population densities in all panels *P*_1_(0) = *P*_2_(0) = 25, *A*(0) = 1. Preferences in (a) are given by the ESS Eq (11). Preferences in (b) and (c) are given by the replicator Eq (4) with *ν* = 1, 0.25 respectively and *u*_1_(0) = 0.999. Parameters as in Table 1, with *K*_*i*_ = 50, *c*_*i*_ = 0.

This is especially easy to observe when there is no direct competition between plants (*c*_1_ = *c*_2_ = 0). Thus, a plant can cause the decrease of the other plant only by influencing pollinator preferences. Let us assume that at time *t* = 0 both plants have equal densities and pollinators are rare (but above the critical Allee threshold density), as shown in Fig 5. Because the plant to pollinator ratio is large, pollinators should specialize on plant 1 (*u*_1_ = 1) which is the most profitable (*e*_1_ > *e*_2_), causing plant 2 to decline and to go extinct, eventually. However, as pollinator densities start to increase relative to plant densities, pollinators can become generalists which favours plant coexistence. We start with the assumption that pollinator preferences track instantaneously population numbers (panel a: *ν* = ∞), i.e., $u_{1}=u_{1}^{*}$ is given by the the ESS Eq (11) (see the star-line -*-*- in Fig 5a). We observe that as pollinator abundance increases, pollinators become generalists approximately at *t* ≈ 3, which is fast enough to prevent plant 2 extinction, and population densities will tend to an interior equilibrium. When pollinators preference is described by the replicator equation (panel b: *ν* = 1), we observe that pollinators will become generalists at a latter time (*t* ≈ 11) due to the time lag with which pollinators preferences follow population abundances. Even with this delay, the decline of plant 2 stops and we obtain convergence to the same population and evolution equilibrium. However, when adaptation is yet slower, the pollinator preferences will follow changing population densities with a longer time delay (panel c: *ν* = 0.25), and we get a qualitatively different outcome with plant 2 extinction. This is because when pollinators start to behave as generalists (*t* ≈ 100), plant 2 abundance is already so low that it is more profitable for pollinators to switch back to pollinate plant 1 only. We obtain similar results as in Fig 5 when e.g., *c*_1_ = *c*_2_ = 0.4, but coexistence becomes impossible when direct competition becomes too strong.

In the next section we study combined effects of initial conditions, plant competition for resources (*c*_*i*_ > 0), and time scales on plant coexistence.

#### Scenario I (variation of initial plant and pollinator densities)

**Fig 6:**
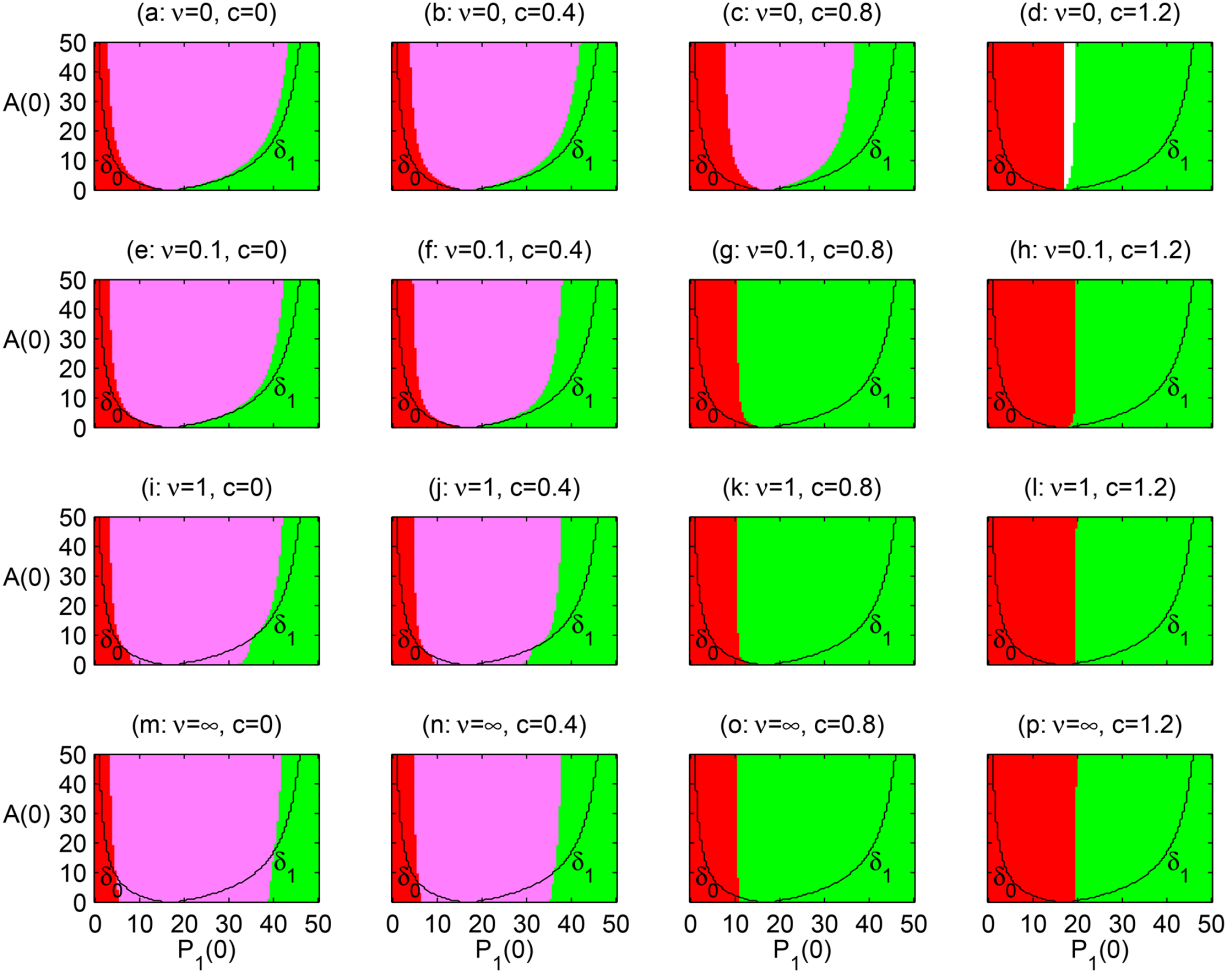
Effects of foraging adaptation (*ν* rows) and inter-specific competition (*c* columns) on plant coexistence under scenario I (variation of initial plant and pollinator densities). *P*_2_(0) = 50 − *P*_1_(0) and *u*_1_(0) given by Eq (11)). Pollinators begin as specialists on plant 1 to right of line *δ*_1_, on plant 2 to the left of line *δ*_0_, and generalists in between. Initial conditions in red and green result in extinction of plant 1 or 2, respectively. Initial conditions in pink and white result in coexistence or community extinction, respectively.

Here we study the combined effects of population dynamics Eq (1a) and adaptive pollinator preferences Eq (4) on species coexistence. Fig 6 shows regions of coexistence (pink), exclusion of one plant species (red or green), and global extinction (both plants and the pollinator, white) for different initial plant and pollinator densities. For this scenario combined initial plant densities are fixed (*P*_1_(0) + *P*_2_(0) = 50). We contrast these predictions with the situation where population densities are fixed, i.e., when population dynamics are not considered and pollinator preferences are at the ESS. In this latter case the necessary condition for both plants to survive is that pollinators behave as generalists which corresponds to the region between the two curves *δ*_0_ and *δ*_1_ in Fig 6. These are the curves along which the ESS predicts switching between specialist and generalist pollinator behaviour at initial population densities. These curves are found by solving Eq (11) for *A*, when $u_{1}^{*}=1$ which yields

$$\begin{array}{c} \delta_{1}\equiv A=\frac{a_{1}e_{1}P_{1}w_{2}}{a_{2}b_{2}e_{2}P_{2}}-\frac{w_{1}}{b_{1}}, \end{array}$$ (12)

and when $u_{1}^{*}=0$ which yields

$$\begin{array}{c} \delta_{0}\equiv A=\frac{a_{2}e_{2}P_{2}w_{1}}{a_{1}b_{1}e_{1}P_{1}}-\frac{w_{2}}{b_{2}}. \end{array}$$ (13)

If population densities do not change, initial conditions to the left (right) of *δ*_0_ (*δ*_1_) lead to exclusion of plant 1 (plant 2) because pollinators specialize on plant 2 (plant 1). Population and pollinator preference dynamics do change these predictions. The main pattern observed in the simulations is that plant coexistence becomes less likely as the plant competition coefficient (*c*) increases. This is not surprising because a higher inter-specific competition between plants decreases plant population abundance which makes coexistence of both plants less likely or impossible (panels c, d, g, h, k, l, o, p). In fact, when inter-specific plant competition is too strong and the pollinators do not adapt, plant densities can become so low that the system collapses due to mutualistic Allee effects (panel d, white region).

The effect of pollinator adaptation rate (*ν*) on coexistence is more complex, in particular when plant competition is moderate or weak (i.e., *c* ≤ 0.4, panels a, b, e, f, i, j, m, n). At low adaptation rates (*ν* ≤ 0.1, panels a, b, e, f) increasing the adaptation rate makes the region of coexistence smaller. With faster adaptation rates (i.e., *ν* ≥ 1, panels i, j, m, n), increasing the adaptation rate further narrows the region of coexistence for large initial pollinator densities, but widens this region for smaller initial pollinator densities (see the pink areas below the *δ*_0_ and *δ*_1_ curves). Although initially pollinators specialize on the more profitable plant, the inter-specific competition among pollinators leads to generalism, and provided the adaptation rate is fast enough, to plant coexistence. This is the same effect as in Fig 5, in which the same set initial conditions with low pollinator densities leads to plant exclusion when adaptation is slow, or coexistence when adaptation is faster.

When competition is strong (*c* = 0.8), the main effect of pollinator adaptation is that coexistence entirely disappears (cf. panel c vs. g, k, o). This is because plant densities are reduced and pollinator densities do not reach high enough densities that would lead to pollinator generalism. Finally, when competition is very strong (*c* = 1.2) global extinctions do not happen (cf. panel d vs. h, l p). This is because adaptation allows pollinators to switch fast enough towards the most profitable plant before competition drives total plant abundance below the Allee threshold that would lead to global extinction.

#### Scenario II (variation on initial plant densities and preferences)

**Fig 7:**
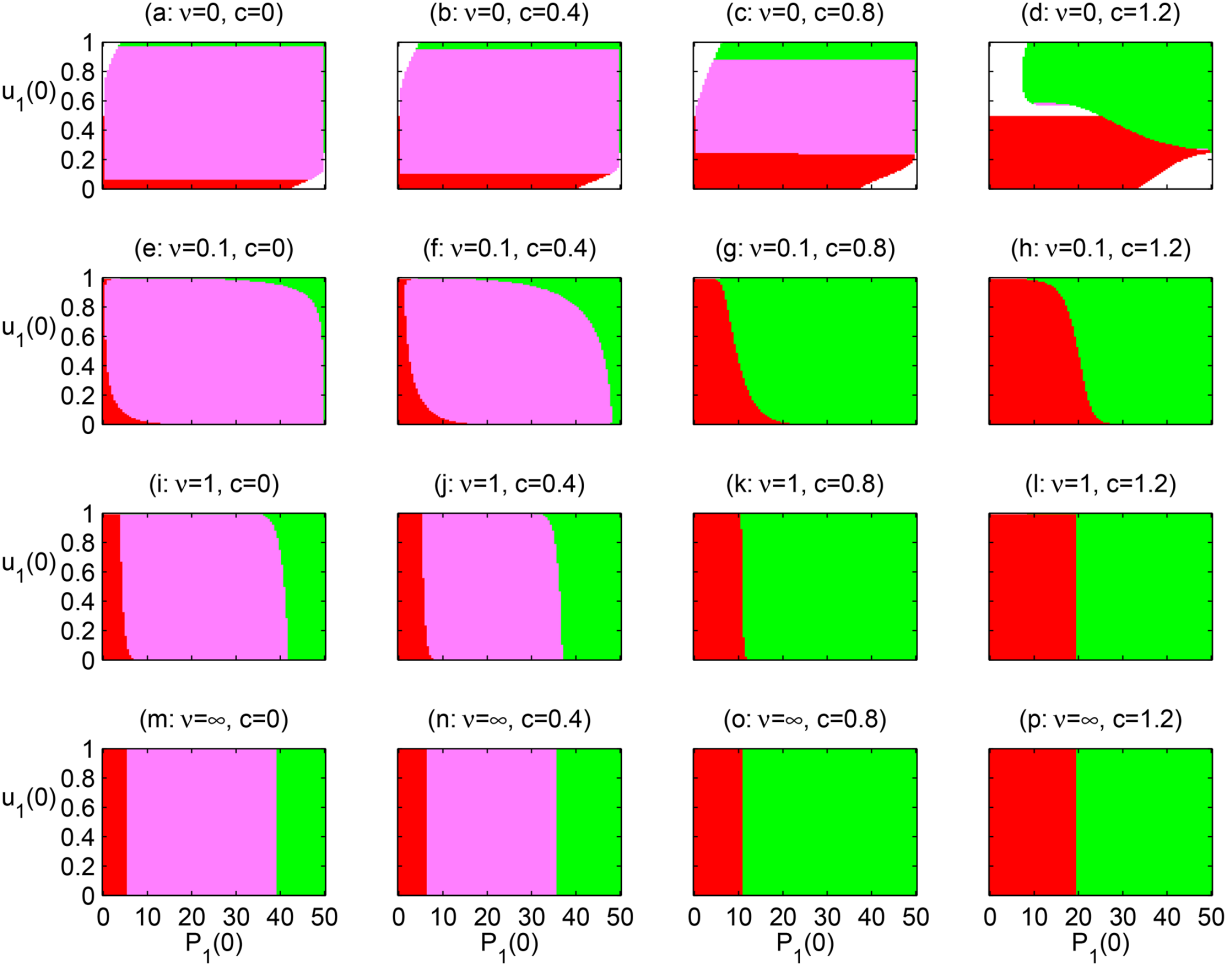
Effects of foraging adaptation (*ν* rows) and inter-specific competition (*c* columns) on plant coexistence under scenario II (variation on initial plant densities and preferences). *P*_2_(0) = 50 − *P*_1_(0) and *A*(0) = 2). Initial conditions in red and green result in extinction of plant 1 or 2, respectively. Initial conditions in pink and white result in coexistence or community extinction respectively.

This scenario focuses on the effect of initial plant population densities and initial pollinator preferences on plant coexistence. Similarly to scenario **I** (Fig 6), increasing the inter-specific plant competition coefficient *c* makes plant coexistence less likely (Fig 7). When pollinators switch from fixed to adaptive foragers the region of plant coexistence becomes smaller (e.g., see the pink region in the first two columns in Fig 7). The general tendency is that increased pollinator adaptation rate reduces the set of initial conditions that lead to coexistence (cf. third vs. second row in Fig 7).

For non-adaptive pollinators, community collapse is more widespread than in the scenario **I** (cf. white regions in Figs 6 vs. 7). This is because in scenario **II** pollinator preferences can initially be extremely biased towards the rarest plant (around the upper-left and bottom-right corners of the panels in Fig 7). These biased initial preferences are obviously maladaptive for the pollinator, but in reality, they can be caused by external disturbances, like the removal of the most preferred plant. For the highest competition level, communities can collapse when both plants are initially abundant and pollinators are generalists (around the centre of panel d). This is caused by the same mechanism outlined for scenario **I**: plants severely harm each other for a long time, causing a critical fall in their combined abundance that leads to extinctions due to the Allee effect. We also observe a very small region where non-adaptive pollinators can coexist with strongly competing plants (the pink region in panel d). We examined the corresponding time series to confirm that they display damped oscillations or limit cycles like in Fig 3b and 3c (results not shown).

Fast enough pollinator adaptation prevents community collapse, by enabling pollinators to abandon initially maladaptive diets before it is too late (cf. the first row vs. e.g., the second row in Fig 7). In the long term either both plants do coexist if plant competition is low (panels e, f, i, j, m, n), or one plant is excluded by the other plant if plant competition is high (panels g, h, k, l, o, p). As the adaptation rate increases, Fig 7 shows an important effect on the general orientation of the regions of coexistence and exclusion. With no adaptation (top row in Fig 7) the outcome (coexistence or exclusion) depends more on the initial preferences (vertical axis) than on the initial plant composition (horizontal axis). However, when adaptation is fast (e.g., third row *ν* = 1), initial preferences have little influence on the outcome (unless grossly biased towards 0 or 1) and initial plant composition is more important. The effect is more sharp when adaptation rate is infinitely fast (*ν* = ∞), because plant coexistence is entirely independent of the initial pollinator preferences.

## Discussion

We studied a two-plant–one-pollinator interaction module assuming that pollinator preferences for plants are either fixed or adaptive. When pollinator preferences are fixed, we observe that for intermediate pollinator preferences, plants can facilitate each other indirectly by raising pollinator densities, thus making coexistence more likely. This effect disappears when preferences are too biased in favour of one plant, or when competition for factors such as resources or space is too strong. While coexistence is predominantly at a population equilibrium, coexistence along a limit cycle is also possible, but under very restrictive conditions in parameter values. Adaptive pollinator foraging introduces additional competition between plants for pollinators, because pollinators switch to the major plants, which is bad for rare plants. This makes coexistence less likely. However, competition for plant resources between pollinators can promote generalism, thus plant coexistence. The net outcome depends on the relative speeds between population dynamics and diet adaptation, the strength of competition between plants for factors other than pollination services, and on the past history of the community (initial conditions).

### Interaction dynamics with fixed preferences

Under fixed pollinator preferences (i.e., no adaptation) Model (1a) reveals a rich set of outcomes. The dynamics are complex because plants and pollinators are obligate mutualists, i.e., their coexistence depends on their population densities being above the Allee (extinction) threshold. Such thresholds become less important when one considers alternative pollination mechanisms (e.g., selfing, wind) or mutualistic partners (other plants, other pollinators), vegetative growth or immigration [25, 27], that our model does not include.

When pollinator preference is extremely biased towards a particular plant, plant coexistence is not possible even when competition for factors such as space or nutrients is not considered. This is because the less preferred plant, being rarely pollinated, cannot increase in abundance. For an intermediate range of pollinator preferences, coexistence is possible through a number of ways. The most simple and familiar is coexistence by mutual invasion, like in the Lotka–Volterra competition model. In this case each plant can attain a positive abundance at an equilibrium with the pollinator in the absence of the other plant, and the missing plant can invade and establish in the community (this happens in the middle “P1 + P2” region of Fig 2a). Another way is when plant *j* can invade the plant *i* and the pollinator community, but plant *j* alone cannot coexist with the pollinator (left or right “P1 + P2” regions in Fig 2a and 2b). In all these cases one plant (*i*) *facilitates* the other plant (*j*) via pollinator sharing, by increasing the pollinator density (see Fig 1b). This indirect, density mediated interaction between plants [7] is called pollinator mediated facilitation [8]. A striking example of pollinator mediated facilitation occurs when neither plant can coexist with the pollinator without the other plant, but pollinators do coexist with both plants (this happens in the “P1 + P2 *or* EXT” region in Fig 2b). However, for trajectories to converge to the interior equilibrium the initial plant densities must be high enough so that the pollinator mediated facilitation is strong enough. Pollinator mediated facilitation [8] has been empirically documented [9, 10], and its role in plant invasions recognized [28].

Invading plants can have positive or negative effects on the resident species. If plant competition is very weak or absent (*c*_*i*_ ≈ 0), the invader can indirectly increase the resident’s plant density. This is another manifestation of pollinator mediated facilitation [8], this time by the invader. If competition is stronger, invasion and establishment can cause decline in the resident plant (e.g., Fig 3a) or its replacement by the invader, as expected according to competition theory [19]. In this case plant competition just outweighs facilitation. Our analysis also shows that in low productive environments (i.e., when Eq (10) does not hold), a resident plant can facilitate the invasion of poor quality plants (with low *e*_*i*_) that cause the subsequent collapse of the whole community (e.g., Fig 3d).

Numerical analysis of Model (1a) shows that when coexistence cannot occur by invasion when rare, it is sometimes achievable if the invader’s density is initially large enough. Coexistence generally takes place at stable densities (e.g., region “P2 *or* P1 + P2” in Fig 2b and most of region “P1 *or* P1 + P2” in Fig 2a). But we also found coexistence along a limit cycle. Limit cycles occur for very narrow ranges of preferences and under strong competition (e.g., a small part of region “P1 *or* P1 + P2” in Fig 2a). We only found limit cycles when low quality plant 2 (*e*_2_ < *e*_1_, Table 1) cannot support the pollinator and cannot invade plant 1–pollinator equilibrium. Only when plant 2 enters at large densities, it will start driving out plant 1, followed by the pollinator. This leads to plant 2 decline and the later recovery of the plant 1–pollinator system, completing the cycle. We could say that such dynamics between plant 1–pollinator subsystem and plant 2 resembles prey–predator or host–parasite interactions. Limit cycles in competitor–competitor–mutualist modules have been predicted before, in models of the Lotka–Volterra type [29]. We never observed limit cycles when pollinator preferences are adaptive (*ν* > 0 in Eq (4)).

We assumed that plant competition affects growth rather than death rates [4, 30]. This assumption is sound when plants are mainly limited by space, or by resources whose access are linked to space, such as light. In such circumstances a plant could produce many seeds thanks to pollination, but space puts a limit on how many will recruit as adults. It remains to see how our results would change if competition is considered differently, when adult plant mortality is affected by competition (e.g., [2]). This can be very important under scenarios of interference like allelopathy or apparent competition caused by herbivores [31].

### Adaptive preferences and population feedbacks

The ESS Eq (11) predicts that when at low densities, pollinators will pollinate only the plant that is most profitable, while at higher densities they will tend to pollinate both plants. This positive relationship between pollinator/consumer abundance and generalism was experimentally demonstrated for bumblebees [13].

Plant and pollinator densities are not static, they change within the limits imposed by several factors: e.g., space and nutrients in the case of plants, or plant resources such as nectar in the case of pollinators. On the other hand, plants and pollinators require minimal critical densities of each other in order to compensate for mortality. Thus, a given ESS at which one plant is excluded from the pollinator’s diet will cause that plant to decrease in density, and, possibly, to go extinct. However, as population densities change the ESS can also change in ways that may favour coexistence. These outcomes will depend on the time scale of pollinator foraging adaptation. For this reason, we introduced the replicator Eq (4) as a dynamic description of pollinator preferences and we coupled it with population dynamics. One of the main consequences of introducing replicator dynamics is the disappearance of complex dynamics such as limit cycles or global extinctions triggered by invasion (Fig 3b and 3d). In contrast, the dynamics with pollinator adaptation are characterized by fewer stable outcomes (plant 1 only, plant 2 only, coexistence) with strong dependence on the initial conditions.

Whether or not adaptive pollinator preferences promote plant coexistence depends critically on the strength of competition (i.e., competition coefficient) and the rate of pollinator adaptation (*ν*). In our simulations, we determined the region of initial population densities and pollinator preferences leading towards plant coexistence, as a function of these two factors. The larger this region, the more likely plant coexistence. As competition strength increases, coexistence becomes less likely as expected from competition theory [19]. As pollinator adaptation rates increase the pattern is more complex and sometimes equivocal, as adaptation can increase or decrease the likelihood of coexistence (Figs 6 and 7). For example, when competition is weak or moderate in simulation scenario **I**, we see that the region of coexistence is generally wider when pollinator densities are initially large and more narrow when pollinators are initially rare (Fig 6 for *c* ≤ 0.4). This agrees with the pattern outlined in Fig 1b and 1c. In other words, when pollinators are abundant competition between pollinators promotes generalism, which is good for plant coexistence, whereas if pollinators are rare they can easily turn into specialists, which is bad for coexistence. However, when the adaptation rate increases, we also observe that if pollinators are initially rare, the region of coexistence widens. Fig 5 can help explain this: a rare pollinator specializes on a single plant, even when neither plant is too rare (e.g., initial ESS panel a). As pollinators start growing, competition for plant resources will cause pollinators to drive towards generalism. If this change is fast enough (large *ν*, panel b) the extinction of the less preferred plant can be prevented. However, if the change is too slow (small *ν*, panel c) the less preferred plant declines too fast to be rescued from extinction. Thus, the results from scenario **I** tells us that time lags in pollinator adaptation with respect to population dynamics have important consequences for plant coexistence.

Simulation scenario **II** also tells us that adaptation lags can affect the entire community, plants and pollinators. In this scenario initial pollinator preferences are arbitrary (i.e., not at the ESS). This is likely to happen if external perturbations (e.g., disease, grazing) makes the most preferred plant too rare and the less preferred too common, in a very short time. If pollinators cannot turn into generalists fast enough they will go extinct because of the mutualistic Allee effect. This leads to the collapse of the community (white regions in Fig 7 at *ν* = 0). When pollinators are able to adapt, such global extinctions can be prevented, sometimes at the price of one plant going extinct. We also observe global extinction in scenario **II** when initial plant ratios and fixed pollinator preferences are both not too biased (i.e., around the centre of panel d in Fig 7). In these particular cases, generalism is not optimal because of splitting foraging effort on both plants, neither plant gets enough pollination services to survive. By adapting its preference towards a single plant (panels h, l, p in Fig 7), the pollinator population would avoid extinction.

Adaptive pollination in a mutualistic interaction module predicts opposite trends for biodiversity when compared with the apparent competition food web module with adaptive consumers. Instead of promoting species coexistence by decreasing competitive asymmetries as in the apparent competition food web module [32–34], adaptive pollinator preferences can increase or decrease plant competitive asymmetries, making their coexistence less or more likely, respectively. At low density, pollinators tend to specialize on the most common plant (Fig 1c), leading to the exclusion of the rare plant. At high density, competition between pollinators promotes generalism (Fig 1d), which helps in promoting plant coexistence. Similar outcomes are predicted in the model studied by Song and Feldman [35], where plant coexistence is favoured under low plant:pollinator ratios (although these ratios were kept fixed by these authors). Because plant:pollinator ratios are dynamic, transitions from specialization to generalism depend not only on adaptation rates, but also on how fast pollinator densities react to simultaneous changes in plant densities. We see this in Fig 5, where pollinators attain large density very quickly, before one plant becomes too common and the other too rare. This indicates that the form of *population regulation* (e.g., linearly decreasing for plants [4, 30] vs. hyperbolically decreasing for pollinators [15]) as well as the *numerical response* towards mutualistic partners (e.g., saturating for plants vs. linear for pollinators [14]), can play important roles in consumer adaptation in mutualistic communities.

Our simulations assume that plant 1 is richer in energy rewards when compared to plant 2 (*e*_1_ > *e*_2_) while we keep all the other plant-specific parameters equal. We also ran simulations with plant 1 being better with respect to other plant-specific parameters (e.g., *r*_1_ > *r*_2_ or *w*_2_ > *w*_1_, keeping *e*_*i*_ = 0.1 and the rest of parameters as in Table 1). In these simulations (not shown here) coexistence is generally more difficult to attain (e.g., coexistence regions as those in Fig 6 get smaller). The reason is that in our model, plant rewards (*e*_*i*_) affect plants only indirectly, by influencing pollinator preferences. In contrast, other plant-specific parameters affect plant dynamics directly. Finally, the larger *c*_*i*_ and *K*_*i*_ the more likely plant *i* always win in competition, but this is a natural result expected in models derived from the Lotka–Volterra competitive equations.

Some predictions from our model are in qualitative agreement with experiments. For example [10] shows transitions from plant facilitation to competition for pollinators [8] when one plant species (*Raphanus raphanistrum*) is exposed to increasing numbers of an alternative plant (*Cirsium arvense*). In the same study, the relative visitation frequency of a plant (*Raphanus*) declines faster than predicted by the decline in the relative proportion of its flowers [10]. The ESS can explain this outcome as the superposition of a relative resource availability effect and a resource switching effect (i.e., first and second terms respectively, in the right-hand-side of Eq (11)), as shown by Fig 4 (compare it with Fig 6 in [10]). The effect of resource competition on the relationship between pollinator density and generalism, was demonstrated by another experiment [13]. Other studies show that invasive plant species can take advantage of changing pollinator preferences, increasing their chances to get included into native communities [36]. Finally, one meta-analysis indicates that pollinators can be taken away by invasive plants, affecting native plants adversely [28].

### Inter-specific pollen transfer effects

Model (1a) considers only one single pollinator species. This makes pollinator generalism (i.e., *u*_1_ strictly between 0 or 1) a requisite for coexistence. However, when pollinators are generalists, rare plants would experience decreasing pollination quality, due to the lack of constancy of individual pollinators delivering non-specific pollen or losing con-specific pollen [37–39]. We do not consider inter-specific pollen transfer effects (IPT) in this article. Modelling IPT effects requires additional assumptions about visitation probabilities [30], pollen carry-over [4] or pollinator structure [35, 40]. Nevertheless, we simulated scenarios **I** and **II** again, but replacing our Eq (1a) by a system of equations that considers IPT [30]. We found that most of our results hold qualitatively, i.e., the coexistence regions display the same patterns like in Figs 6 and 7 (results not shown).

There is no question that IPT affects pollination efficiency. However, the relative importance of IPT may also depend on the structure of the environment where interactions occur. A survey of field and laboratory results [39] reports that in spite of strong IPT effects on plant reproduction for certain systems, many studies found little or no significant effects in other systems. One reason could be the scale of the system under study, which can influence the way mobile pollinators experience the resource landscape: fine grained or coarse grained, e.g., well mixed or patchy. Thus, if plant species are not totally intermingled, but also not isolated in clumps, the negative effects of IPT on seed set (a proxy for plant fitness) could be reduced [41]. In addition, unless we consider a single flower per plant at any time, the resource is almost always patchy. This means that IPT effects in self-compatible plants would be stronger just after pollinator arrival, decreasing for the remaining flowers before the pollinator leaves the plant.

### From modules to networks and from adaptation to co-evolution

The scope of our work is limited to adaptation in a single pollinator species only. In real life settings adaptation can be affected by (i) competition among several pollinator species, and by (ii) plant–pollinator co-evolution.

With respect to point (i), large community simulations [30] indicate that inter-specific competition can force pollinators to change their preferences in order to minimise niche overlap. This can promote coexistence and specialization on rare plants at risk of competitive exclusion. Song and Feldman [35] discovered a similar mechanism, with a polymorphic pollinator, i.e., consisting of specialist and generalist sub-populations. Thus, adding a second pollinator would be a next step to consider, in order to address inter-specific competition.

Addressing point (ii) will require trade-offs in plant traits. We showed how differences in pollinator efficiencies (*e*_*i*_) indirectly affect plant dynamics Eq (1a). However, pollinator efficiencies can depend on plant allocation patterns, which can affect their growth, mortality or competitive performance (*r*_*i*_, *m*_*i*_, *c*_*i*_). Plant adaptation likely happens over generations, so a replicator equation approach or adaptive dynamics [22] will be useful to study plant–pollinator co-evolution.

In spite of the complexity of real plant pollinator networks, small community modules will remain useful to tease apart the mechanisms that regulate their diversity. Models of intermediate complexity like Eq (1a) can help us discover important results concerning interaction dynamics, pollinator foraging patterns (e.g., pollinator ESS) and the consequences of differences between ecological vs. adaptation time scales.

## Supporting Information

S1 Appendix Analysis with fixed preferences. (PDF)

S1 File Source codes used to generate figures and simulations. (ZIP)
