# Supplementary material for: Pollinator Foraging Adaptation and Coexistence of Competing Plants
Source: PLoS One. 2016 Aug 9;11(8):e0160076. doi: 10.1371/journal.pone.0160076 (PMC4978411; doi:10.1371/journal.pone.0160076)
Supplement: S1 Appendix — (PDF) [file pone.0160076.s001.pdf]

Supporting Information of
“Pollinator foraging flexibility and coexistence of competing plants”

Tomas A. Revilla & Vlastimil Křivan

S1 Analysis with fixed preferences

The community model used in the main text can be derived from a mass action mechanism that considers plant resource dynamics explicitly[1]

$$\frac{dF_i}{dt} = a_i P_i - w_i F_i - u_i b_i F_i A \tag{S.1a}$$

$$\frac{dP_i}{dt} = \begin{cases} r_i u_i b_i F_i A \left(1 - \frac{P_i + c_j P_j}{K_i}\right) - m_i P_i & \text{if } P_i > 0 \\ 0 & \text{if } P_i = 0 \end{cases} \tag{S.1b}$$

$$\frac{dA}{dt} = (e_1 u_1 b_1 F_1 + e_2 u_2 b_2 F_2 - d)A, \tag{S.1c}$$

where $i, j = 1, 2$ with $i \neq j$. In this model F_i denotes density of plant i resources such as nectar. Note that pollinator birth rates are directly proportional to plant resources, like in most consumer–resource models. Plant birth rates are proportional to the product between plant resource and pollinator densities, on the assumption that the rate of plant pollination relates linearly with the rate of pollinator resource consumption. In (S.1b) we prevent plant i to reach negative densities by setting their population growth to zero when there is no plant i. Next, we assume that resources equilibrate quickly with current plant and pollinator densities (i.e., $dF_i/dt = 0$, while $dP_i/dt \neq 0$ and $dA/dt \neq 0$). Thus $F_i = a_i P_i/(w_i + u_i b_i A)$, which we substitute in (S.1b) and (S.1c), to get the system of ordinary differential equations (ODE) shown in the main text as “(1)”. Note that the ODE system in the main text keeps the positive octant invariant (i.e., non-negative) so we do not need any additional assumption on plant growth when at zero density. Our analysis is much easier to follow if we re-arrange the ODE in a form that resembles classical competition (Lotka–Volterra) and consumer–resource models

$$\begin{aligned} \frac{dP_1}{dt} &= g_1(A)\left(1 - \frac{P_1 + c_2 P_2}{k_1(A)}\right) P_1 \\ \frac{dP_2}{dt} &= g_2(A)\left(1 - \frac{P_2 + c_1 P_1}{k_2(A)}\right) P_2 \\ \frac{dA}{dt} &= \left(e_1 h_1(A) P_1 + e_2 h_2(A) P_2 - d\right) A \end{aligned} \tag{S.2}$$

with

$$g_i(A) = \frac{r_i a_i u_i b_i A}{w_i + u_i b_i A} - m_i \tag{S.3}$$

$$k_i(A) = K_i \left(1 - \frac{m_i(w_i + u_i b_i A)}{r_i a_i u_i b_i A}\right) \tag{S.4}$$

$$h_i(A) = \frac{a_i u_i b_i}{w_i + u_i b_i A}. \tag{S.5}$$

[1] Revilla, T. A. (2015) Numerical responses in resource-based mutualisms: a time scale approach, Journal of Theoretical Biology, 378:39–46.

The plant intrinsic growth rates $g_i(A)$ and the carrying capacities $k_i(A)$ are saturating functions of pollinator density, i.e., $\lim_{A\to\infty} g_i(A) = r_i a_i - m_i$ and $\lim_{A\to\infty} k_i(A) = K_i(1 - m_i/(r_i a_i))$. Pollinator per capita consumption rates $h_i(A)$ decrease to 0 with increasing pollinator density due to intra-specific pollinator competition for plant resources. We observe that at low pollinator densities both g_i and k_i are negative.

System (S.2) has the extinction equilibrium $(P_1, P_2, A) = (0, 0, 0)$. The Jacobian matrix evaluated at this equilibrium is

$$J(0,0,0) = \begin{bmatrix} -m_1 & 0 & 0 \\ 0 & -m_2 & 0 \\ 0 & 0 & -d \end{bmatrix}. \tag{S.6}$$

Thus, all eigenvalues are negative and the trivial equilibrium is locally asymptotically stable. There are also other, non-trivial equilibria that we consider next.

S1.1 Single plant–pollinator equilibria

Let us assume that plant 2 is absent and we study the plant 1–pollinator subsystem. By setting $P_2 = 0$ the nullcline of plant 1 is

$$P_1 = k_1(A) = K_1 \left(1 - \frac{m_1(w_1 + u_1 b_1 A)}{r_1 a_1 u_1 b_1 A}\right), \tag{S.7}$$

see Figure S.1. The plant nullcline crosses the A axis at

$$A_1^* = \frac{m_1 w_1}{u_1 b_1 (r_1 a_1 - m_1)} \tag{S.8}$$

and it has a vertical asymptote at

$$P_1 = K_1 \left(1 - \frac{m_1}{r_1 a_1}\right). \tag{S.9}$$

The plant 1 nullcline is in the positive quadrant of the plant 1–pollinator phase space provided

$$r_1 a_1 > m_1. \tag{S.10}$$

Setting $P_2 = 0$ in (S.2) we get the pollinator nullcline

$$P_1 = \frac{d}{e_1 h_1(A)} = \frac{d(w_1 + u_1 b_1 A)}{e_1 a_1 u_1 b_1} \tag{S.11}$$

which crosses P_1 axis at

$$P_1^* = \frac{d w_1}{e_1 a_1 u_1 b_1}. \tag{S.12}$$

Figure S.1 shows two possible nullcline configurations. Provided preference for plant 1 is strong enough and satisfies

$$u_1 > u_{1a} = \frac{d r_1 w_1}{b_1 e_1 (\sqrt{a_1 r_1} - \sqrt{m_1})^2 K_1}, \tag{S.13}$$

the nullclines intersect at two positive equilibria (Panel b) (P_{1-}, A_{1-}) and (P_{1+}, A_{1+}) where

$$P_{1\pm} = \frac{b_1 e_1 K_1 (a_1 r_1 - m_1) u_1 + d r_1 w_1 \pm \sqrt{D_1}}{2 a_1 b_1 e_1 r_1 u_1}$$

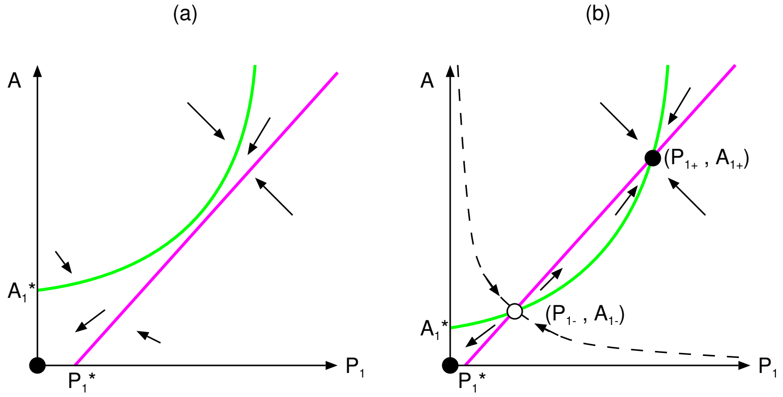

Figure S.1. Plant 1 and pollinator phase plane. The plant and pollinator (non trivial) nullclines are coloured green and pink respectively. (a) When (S.13) does not hold the nullclines don't intersect and thus both species go extinct. (b) When (S.13) holds the nullclines intersect at two equilibrium points: a saddle point which is unstable (circle) and a locally stable node (dot). Plant and pollinator coexist for combinations of densities above the separatrix passing through the saddle point (dash line).

$$A_{1\pm} = \frac{b_1 e_1 K_1 (a_1 r_1 - m_1) u_1 - d r_1 w_1 \pm \sqrt{D_1}}{2 b_1 d r_1 u_1},$$

and

$$D_1 = -4 b_1 d e_1 K_1 m_1 r_1 u_1 w_1 + (b_1 e_1 K_1 (m_1 - a_1 r_1) u_1 + d r_1 w_1)^2. \tag{S.14}$$

When u_1 does not meet the threshold in (S.13), no positive interior equilibrium exists (Panel a).

The Jacobian matrix evaluated at one of these two interior equilibria (i.e., $(P_1, A) = (P_{1+}, A_{1+})$ or $(P_1, A) = (P_{1-}, A_{1-})$) is

$$J(P_1, A) = \begin{bmatrix} -\frac{A a_1 b_1 P_1 r_1 u_1}{K_1 (A b_1 u_1 + w_1)} & \frac{a_1 b_1 (K_1 - P_1) P_1 r_1 u_1 w_1}{K_1 (A b_1 u_1 + w_1)^2} \\ \frac{A a_1 b_1 e_1 u_1}{A b_1 u_1 + w_1} & -\frac{A a_1 b_1^2 e_1 P_1 u_1^2}{(A b_1 u_1 + w_1)^2} \end{bmatrix}. \tag{S.15}$$

We observe that the trace of the Jacobian is negative and the determinant is

$$\det(J) = \frac{A a_1^2 b_1^2 e_1 P_1 r_1 u_1^2 (A b_1 P_1 u_1 + (P_1 - K_1) w_1)}{K_1 (A b_1 u_1 + w_1)^3}.$$

For an interior equilibrium to be locally asymptotically stable, the determinant must be positive, i.e.,

$$P_1 > \frac{K_1 w_1}{w_1 + A b_1 u_1}.$$

Substituting the two interior equilibria into this inequality, it is easy to see that only the equilibrium with the higher plant density (P_{1+}, A_{1+}) satisfies the above inequality and it is therefore locally stable, while the other equilibrium is unstable. The position of the two nullclines in Figure S.1b confirms that (P_{1+}, A_{1+}) is a stable node and (P_{1-}, A_{1-}) is a saddle point.

All the results from this section are valid if we ignore plant 1 instead of plant 2, by changing the sub-index 1 to 2. We note that the equilibria $(P_{2\pm}, A_{2\pm})$

$$P_{2\pm} = \frac{b_2 e_2 K_2 (a_2 r_2 - m_2) u_2 + d r_2 w_2 \pm \sqrt{D_2}}{2 a_2 b_2 e_2 r_2 u_2}$$

$$A_{2\pm} = \frac{b_2 e_2 K_2 (a_2 r_2 - m_2) u_2 - d r_2 w_2 \pm \sqrt{D_2}}{2 b_2 d r_2 u_2},$$

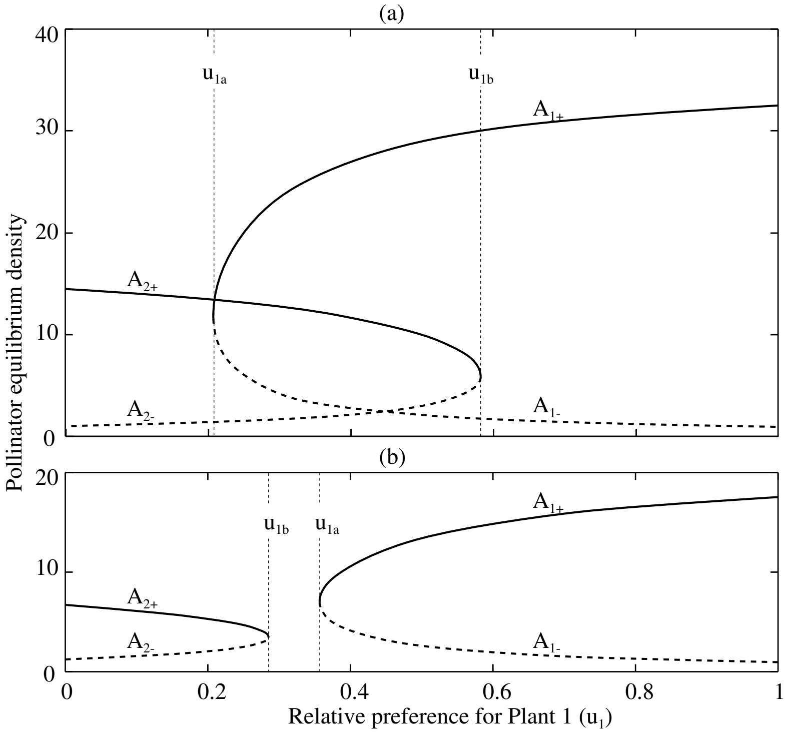

Figure S.2. Bifurcation plots displaying stable (A_{i+}, solid line) and unstable (A_{i-}, dash line) pollinator equilibria under coexistence with plant $i = 1$ or $i = 2$ alone. (a) For $K_1 = K_2 = 60$ inequality (S.18) holds. (b) For $K_1 = K_2 = 35$ inequality (S.18) does not hold. The rest of the parameters are as in Table 1 from the main text.

and

$$D_2 = -4b_2de_2K_2m_2r_2u_2w_2 + (b_2e_2K_2(m_2 - a_2r_2)u_2 + dr_2w_2)^2. \tag{S.16}$$

exist provided

$$u_1 < 1 - \frac{dr_2w_2}{b_2e_2(\sqrt{a_2r_2} - \sqrt{m_2})^2K_2} = u_{1b}. \tag{S.17}$$

Combining (S.13) and (S.17) we observe that when the environmental carrying capacity for plant 1 is large so that

$$K_1 > K_1^* = \frac{b_2de_2K_2r_1(\sqrt{m_2} - \sqrt{a_2}\sqrt{r_2})^2w_1}{b_1e_1(\sqrt{m_1} - \sqrt{a_1}\sqrt{r_1})^2(b_2e_2K_2(\sqrt{m_2} - \sqrt{a_2}\sqrt{r_2})^2 - dr_2w_2)} \tag{S.18}$$

the two equilibria (P_{1+}, A_{1+}) and (P_{2+}, A_{2+}) can coexist when $u_{1a} < u_1 < u_{1b}$. If inequality (S.18) is reversed, then $u_{1a} > u_{1b}$ and both single-plant–pollinator equilibria cannot coexist. Figure S.2 shows the dependency of both equilibria, when carrying capacities are large and small. Numerical bifurcation analysis indicates that equilibria always come as pairs, an unstable low density equilibrium and a locally stable high density equilibrium (Fig. S.1b).

Because

$$\frac{\partial}{\partial P_1}\left(\frac{1}{P_1A}g_1(A)\left(1 - \frac{P_1}{k_1(A)}\right)P_1\right) + \frac{\partial}{\partial A}\left(\frac{1}{P_1A}\left(e_1h_1(A)P_1 - d\right)A\right) =$$

$$-\frac{(a_1b_1u_1(b_1(e_1K_1 + Ar_1)u_1 + r_1w_1)}{K_1(Ab_1u_1 + w_1)^2)}$$

is negative, the Dulac criterion[2] implies that no limit cycles involving only plant 1 (or plant 2) and the pollinator exist.

S1.2 Two plant–pollinator coexistence by invasion

Numerical analysis shows there is also a locally stable interior equilibrium at which both plants coexist with pollinators. Unfortunately, this equilibrium cannot be expressed in a closed form and must be analysed numerically. Invasion analysis provides some partial insight in conditions for species coexistence.

We start with the case where one plant species coexists with pollinators at the interior locally stable equilibrium and we ask under which conditions the missing plant can invade. Let us consider the equilibrium $(P_{1+}, 0, A_{1+})$ at which plant 2 is missing. This equilibrium exists provided inequality (S.13) holds. In Figure S.2 the region of parameters where this equilibrium exists is to the right of the vertical line at u_{1a}. Invasibility of the missing plant 2 requires

$$g_2(A_{1+})\left(1 - \frac{c_1 P_{1+}}{k_2(A_{1+})}\right) > 0, \tag{S.19}$$

i.e., both $g_2(A_{1+})$ and $(1 - c_1 P_{1+}/k_2(A_{1+}))$ must have the same sign. Because $g_2(A)$ and $k_2(A)$ have the same sign for all positive A's it follows that if g_2 in (S.19) is negative, the second term in parentheses must be positive and (S.19) cannot hold. Consequently, the invasion rate can be positive only if $g_2(A_{1+})$ is positive, i.e., when the pollinator abundance at the plant 1–pollinator population equilibrium is high enough and satisfies

$$A_{1+} > \frac{m_2 w_2}{u_2 b_2 (r_2 a_2 - m_2)} \tag{S.20}$$

to ensure plant 2 positive invasion growth rate. From (S.8) we can see that the right-hand-side of this inequality is the threshold pollinator density A_2^*. In other words, invasion requires that the pollinator density at the equilibrium $(P_{1+}, 0, A_{1+})$ must be higher than the minimum mutualistic requirement of the invader (A_2^*).

Provided (S.20) holds, the second term in the right-hand-side of (S.19) is positive if

$$c_1 P_{1+} < k_2(A_{1+}), \tag{S.21}$$

i.e., plant 1 equilibrium density cannot be too high to prevent invasion of plant 2, due to strong competition. Substituting the values of P_{1+} and A_{1+} in this inequality, we obtain an inequality in the form $c_1 < \alpha(u_1)$, where

$$\alpha(u_1) = \frac{a_1 b_1 r_1 u_1 K_2 \left(2 b_2 u_2 e_1 K_1 m_1 w_1 (a_2 r_2 - m_2) - m_2 w_2 \left(b_1 e_1 K_1 u_1 (a_1 r_1 - m_1) - d r_1 w_1 - \sqrt{D_1}\right)\right)}{a_2 b_2 r_2 u_2 K_1 m_1 w_1 \left(b_1 e_1 K_1 u_1 (a_1 r_1 - m_1) + d r_1 w_1 + \sqrt{D_1}\right)}, \tag{S.22}$$

with D_1 given by (S.14).

Similarly, we obtain invasibility conditions for plant 1 to invade plant 2–pollinator stable interior equilibrium

$$A_{2+} > \frac{m_1 w_1}{u_1 b_1 (r_1 a_1 - m_1)}$$

and

$$c_2 P_{2+} < k_1(A_{2+}). \tag{S.23}$$

Substituting P_{2+} and A_{2+} in the inequality above, we obtain an inequality in the form $c_2 < \beta(u_1)$, where

[2] J. Hofbauer and K. Sigmund (1998) Evolutionary Games and Population Dynamics, Cambridge University Press.

$$\beta(u_1) = \frac{a_2 b_2 r_2 u_2 K_1 \left(2 b_1 u_1 e_2 K_2 m_2 w_2 (a_1 r_1 - m_1) - m_1 w_1 \left(b_2 e_2 K_2 u_2 (a_2 r_2 - m_2) - d r_2 w_2 - \sqrt{D_2}\right)\right)}{a_1 b_1 r_1 u_1 K_2 m_2 w_2 \left(b_2 e_2 K_2 u_2 (a_2 r_2 - m_2) + d r_2 w_2 + \sqrt{D_2}\right)}. \tag{S.24}$$

In the parameter space showed in the main text, the graph of $\alpha(u_1)$ is to the right of the u_{1a} vertical line. Below α and right of u_{1a} plant 2 can invade plant 1. And the graph of $\beta(u_1)$ is to the left of the u_{1b} vertical line. Below β and left of u_{1b} plant 1 can invade plant 2. Numerical results indicate that when both plants can invade each other, i.e., when $u_{1a} < u_1 < u_{1b}$, $c_1 < \alpha$ and $c_2 < \beta$, both plants and the pollinator attain a locally stable equilibrium. In other words we get confirmation that mutual invasibility implies stable coexistence. However, when mutual invasibility does not hold, e.g., when only one plant can be a resident, numerical results indicate more complicated outcomes (see main text).

Because $P_{1+} = k_1(A_{1+})$ and $P_{2+} = k_2(A_{2+})$, conditions (S.21) and (S.23) imply that

$$c_1 c_2 < Q = \frac{k_2(A_{1+})}{k_1(A_{1+})} \frac{k_1(A_{2+})}{k_2(A_{2+})}. \tag{S.25}$$

This inequality is similar to the competitive exclusion principle[3] which states that two competing species can coexist only when $c_1 c_2 < 1$, i.e., when the inter-specific competition is weaker when compared to intra-specific competition. In the above inequality the right-hand-side (Q) is not equal to 1, but it depends on the pollinator densities in both single-species–pollinator equilibria. Thus, this inequality generalises the competitive exclusion principle to a mutualistic–competitive system with two plants sharing a pollinator.

[3]Gause, G. F. (1934) The Struggle for Existence, Williams & Wilkins.
